# Evaluation of the immunoprotective potential of recombinant EtMIF as a subunit vaccine candidate against *Eimeria tenella* infection in chickens

**DOI:** 10.3389/fimmu.2025.1623743

**Published:** 2025-07-11

**Authors:** Rui Bai, Hui Wang, Jiale Guo, Yang Pei, Yongbin Li, Shuying Zhu, Chenyang Lv, Jianhui Li, Xiaozhen Cui, Xiaoling Lv

**Affiliations:** ^1^ College of Veterinary Medicine, Shanxi Agricultural University, Jinzhong, China; ^2^ Shanxi Key Laboratory for Modernization of Traditional Chinese Veterinary Medicine TCVM, College of Veterinary Medicine, Shanxi Agricultural University, Jinzhong, China; ^3^ Yakeshi City Animal Disease Prevention and Control Center, Hulunbuir, China; ^4^ College of Animal Science, Shanxi Agricultural University, Jinzhong, China

**Keywords:** *Eimeria tenella*, recombinant EtMIF protein, immunoprotective, expression changes, protein vaccine

## Abstract

**Introduction:**

Eimeria tenella is recognized as the most pathogenic species of chicken coccidia. Infection with E. tenella results in digestive disorders and hemorrhagic diarrhea in chickens. Furthermore, E. tenella has recently shown high incidence and mortality rates. Therefore, developing effective vaccines is vital for controlling this disease. Macrophage migration inhibitory factor (MIF) is recognized as a key upstream cytokine that mediates innate and adaptive immune responses, drawing significant attention.

**Methods:**

In this study, we amplified the E. tenella MIF (EtMIF) gene sequence, constructed the pET-28a-EtMIF prokaryotic expression vector, and expressed and purified the recombinant EtMIF (rEtMIF) protein. The rEtMIF protein localization was determined using immunofluorescence staining, and its immunoprotective efficacy at three different doses (50 µg, 100 µg, and 150 µg) was subsequently evaluated through animal trials.

**Results:**

The rEtMIF protein was approximately 12 kDa in size and primarily existed in a soluble form. The optimal induction conditions were 37°C for 4 hours, and the optimal imidazole elution concentration was 500 mmol/L. The rEtMIF protein was recognized by 6×His-tagged monoclonal antibodies, infection-positive chicken serum, and rabbit anti-rEtMIF polyclonal antibodies. Indirect immunofluorescence analysis demonstrated that the rEtMIF protein was localized both on the surface and within the merozoites of E. tenella. Evaluation of immune protection showed that weight gain in the immunized groups was significantly higher than in the non-immunized group (P < 0.05). Additionally, intestinal lesion scores and oocyst output were significantly reduced (P < 0.05). Among all groups, the 50 µg rEtMIF group achieved the highest anticoccidial index (ACI) value of 161.48. Levels of serum antibodies and cytokines, including IL-1, IL-8, IFN-γ, and TNF-α, were significantly elevated in the immunized groups, indicating that recombinant rEtMIF can stimulate both humoral and cellular immune responses in chickens.

**Discussion:**

This study support the potential of recombinant rEtMIF as a promising candidate for developing vaccines against chicken coccidiosis.

## 1 Introduction

Chicken coccidiosis is a parasitic protozoan disease caused by infection with one or more species of *Eimeria* that target the intestinal epithelial cells of chickens, leading to dysentery, malnutrition, and mortality (1). This disease significantly affects the growth, development, and productivity of chickens, resulting in estimated global economic losses of approximately £10.4 billion annually (2). Among the various *Eimeria* species, *Eimeria tenella* is recognized as the most pathogenic and widespread, primarily infecting the cecum and causing severe economic damage (3).

Currently, chemical anticoccidial drugs remain the primary method for treating and preventing chicken coccidiosis. However, the emergence of drug-resistant strains not only compromises the immune system of chickens but also leads to the accumulation of drug residues and elevated treatment costs (4). Although traditional vaccines based on virulent and precocious strains have been used, their application is hampered by high production costs, labor-intensive manufacturing processes, and the risk of reversion to virulence, ultimately resulting in outbreaks of coccidiosis (5). Therefore, developing a safe, effective, low-toxicity, and environmentally friendly vaccine represents a pivotal strategy toward the sustainable management of chicken coccidiosis. Recent studies have indicated that recombinant protein vaccines offer a promising solution to this challenge by providing targeted, safe, and efficient immunoprotection (6).

Macrophage migration inhibitory factor (MIF) is a pro-inflammatory and immunomodulatory cytokine with tautomerase and oxidoreductase activities (7). It has been demonstrated that MIF participates in both innate and adaptive immune responses by stimulating the production of pro-inflammatory mediators such as TNF-α and IL-8 (8). Moreover, MIF has been shown to maintain macrophage survival and pro-inflammatory functions by inhibiting activation-induced, p53-dependent apoptosis (9). Cytokines regulated by MIF are critically involved in the pathogenesis of various infectious and non-infectious inflammatory diseases.

Miska et al. (10) first identified MIF homologs in the apicomplexan parasites *E. acervulina* and *E. tenella*. The expression of *Eimeria MIF* mRNA was high in merozoites, low in both unsporulated and sporulated oocysts, and nearly undetectable in sporozoites. Kim’s study further demonstrated that the *MIF* genes from chickens and *E. acervulina* can bind to chicken monocytes/macrophages via the surface receptor CD74p41, thereby inhibiting random migration, inducing pro-inflammatory cytokines, and modulating Th1/Th2 cytokine profiles in antigen-presenting immune cells (11). Homologs of MIF have been identified in several parasitic species, including *Toxoplasma gondii*, *Plasmodium*, and *Leishmania* spp (12, 13)., where they promote host immune responses during infection, suggesting that MIF homologs in parasites may be crucial in modulating host immune responses.

Given the critical role of MIF in immune regulation, we hypothesize that the *E. tenella* MIF (EtMIF) homolog may serve as a promising subunit vaccine candidate against *E. tenella* infection. Therefore, this study aims to evaluate the immune protective efficacy of recombinant EtMIF (rEtMIF) against chicken coccidiosis and to identify vaccine candidate genes with strong immunoprotective potential, thereby providing a theoretical and experimental foundation for developing immune-based strategies for the prevention and control of coccidiosis. This research has significant theoretical and practical value for advancing the prevention of coccidiosis and promoting poultry health management.

## 2 Materials and methods

### 2.1 Experimental animals and parasites

14-day-old specific-pathogen-free (SPF) White Leghorns were purchased from Beijing Boehringer Ingelheim Vital Biotechnology Co., Ltd. (Beijing, China) and housed in coccidia-free isolators under controlled conditions. All chicks were reared under SPF conditions with ad libitum access to food and tap water. The basal grower diet was based on Crude protein (≥19.5%), Crude fiber (≤5.5%), Crude ash (≤8.0%), Calcium (0.80~1.20%), Total phosphorus (≥0.30%), Sodium chloride (0.30~0.80), and Methionine(0.45~0.90%). Two-month-old New Zealand White rabbits were obtained from Beijing Baierkangnate Experimental Rabbit Breeding Biotechnology Development Co., Ltd. (Beijing, China). All rabbits were screened for *Eimeria* infection by three consecutive days of fecal examination and subsequently maintained under coccidia-free conditions. Throughout the experiment, animals were monitored daily by veterinarians for general health status, including feed and water intake, behavior, posture, and any signs of clinical symptoms such as diarrhea, weight loss, lethargy, or signs of distress. Animals that reached predetermined humane endpoints were humanely euthanized to minimize suffering. The Shanxi virulent strain of *E. tenella* (SX-01) was propagated and maintained via cecal passage in SPF chickens at the Veterinary Pathology Laboratory of the College of Veterinary Medicine, Shanxi Agricultural University.

### 2.2 Collection of *E. tenella* merozoites and cDNA synthesis from the isolated merozoites

The collection of merozoites was performed according to previously described protocols (14, 15). Briefly, ten 20-day-old SPF chickens were orally inoculated with 1 × 10^4^ oocysts each. At 120 hours post-infection, the chickens were euthanized via CO_2_ asphyxiation. Caecal tissues were collected, finely minced, and incubated with hyaluronidase (Solarbio, Shanghai, China) at 37°C for 60 min. The resulting mixture was filtered and centrifuged at 3000 rpm for 5 min to collect the precipitate. The pellet was incubated with red blood cell lysis buffer (Beyotime, Shanghai, China) at 4°C for 10 min, and merozoites were subsequently purified by density gradient centrifugation.

Total RNA was extracted from *E. tenella* merozoites using TRIzol reagent ((Invitrogen, Carlsbad, CA, USA) according to the manufacturer’s instructions. Subsequently, cDNA was synthesized using the PrimeScript™ RT reagent kit with gDNA Eraser (TaKaRa, Shiga, Japan), according to the manufacturer’s instructions for genomic DNA removal and reverse transcription. The gDNA removal step was conducted at 42°C for 2 min, followed by reverse transcription at 37°C for 15 min and 85°C for 5 s. The resulting cDNA was stored at –80°C until further use (16).

### 2.3 Construction of recombinant pasmid

Specific primers were designed to amplify the *E. tenella MIF* (*EtMIF*) gene sequence based on GenBank ID DQ323515.1, and were synthesized by Suzhou Jinweizhi Biotechnology Co., Ltd. (Suzhou, China). The primer sequences were as follows: *EtMIF* forward primer, 5’-GCTCGAGTTAACCAAACACGCGGGAACC-3’; and reverse primer, 5’-GGCTAGCATGCCACTGTGCCAGATCG-3’. PCR products were purified and cloned into the pMD18-T vector (Takara, Shiga, Japan) according to the manufacturer’s instructions for the gel extraction kit. A single colony was selected and cultured for plasmid extraction and sequencing. The colonies with correct insertions were chosen for subsequent experiments. The *EtMIF* gene fragment was amplified by RT-PCR from RNA isolated from merozoites of *E. tenella* (Shanxi virulent strain). The pMD18-T-EtMIF plasmid and the pET-28a(+) vector (Sangon Biotech, Shanghai, China) were digested with *NheI* and *XhoI* restriction enzymes (Takara, Shiga, Japan) and purified. The *EtMIF* gene fragment was then ligated into the pET-28a(+) vector, and the recombinant plasmid was confirmed by DNA sequencing. The plasmid with the correct sequence was designated pET-28a(+)-EtMIF.

### 2.4 Protein expression and purification

The recombinant pET-28a(+)-EtMIF plasmids were transformed into Escherichia coli BL21 (DE3) competent cells (TransGen, Beijing, China). Single colonies were selected and cultured in LB medium (Solarbio, Beijing, China) supplemented with 50 μg/mL kanamycin at 37°C and 170 rpm until reaching the mid-logarithmic phase (approximately 1 h). The culture was then inoculated into 500 mL of fresh LB medium (Solarbio, Beijing, China) supplemented with 50 μg/mL kanamycin and incubated at 37°C with shaking at 200 rpm. When the OD600 of the culture reached approximately 0.5, isopropyl β-D-1-thiogalactopyranoside (IPTG; Solarbio, Beijing, China) was added to a final concentration of 1 mM to induce protein expression.

Following IPTG induction, the bacterial cells were harvested by centrifugation and lysed by sonication on ice. The lysate was centrifuged to separate the soluble and insoluble fractions. The rEtMIF protein was purified using Ni-NTA affinity chromatography (Sangon, Shanghai, China), The Ni-NTA column was pre-equilibrated with 5 mL of deionized water and 5 mL of binding buffer (20 mM Tris, 500 mM NaCl, 20 mM imidazole, pH 8.0), after which the supernatant was slowly loaded onto the column. After washing with 3 mL of binding buffer, the column was sequentially eluted with 3 mL volumes of elution buffer containing increasing concentrations of imidazole (50, 100, 150, 200, and 500 mM). The collected elution fractions were dialyzed stepwise against PBS to remove imidazole, with the buffer replaced every 4 h over a 12-hour period. To further concentrate the sample, the dialyzed protein was centrifuged in 3 kDa ultrafiltration tubes at 6000 rpm for 40 min at 4 °C and reduced to a final volume of approximately 200 μL. Endotoxin in the purified rEtMIF protein was removed using a Protein Endotoxin Removal Kit (Beyotime, Shanghai, China), and residual endotoxin levels were measured using a Chromogenic LAL Endotoxin Assay Kit (Beyotime, Shanghai, China). Its molecular weight was assessed by 12% SDS-PAGE. Additionally, its concentration was quantified using a BCA Protein Assay Kit (Beyotime, Shanghai, China). The purified rEtMIF protein was aliquoted and stored at −80°C for subsequent experiments (17). Rigorous measures were implemented to minimize the risk of contamination during protein preparation, including ultraviolet irradiation, high-temperature treatment, and the use of sterile, nuclease-free, and endotoxin-free consumables.

### 2.5 Preparation of anti-*E. tenella* and anti-rEtMIF positive serum

During the experiment, 1×10^4^ sporulated *E. tenella* oocysts were orally administered to 20-day-old SPF chickens via crop instillation. Each chick was subsequently challenged with 1×10^4^ sporulated oocysts every 7 days for a total of four times. Blood samples were collected via cardiac puncture, incubated at 37°C for 1 h and then at 4°C for 12 h. Samples were centrifuged at 3000 rpm for 10 min, and serum was separated to prepare anti-*E. tenella* positive serum. Serum was aliquoted and stored at −80°C until use. Serum from uninfected rabbits was collected as a negative control.

Three two-month-old New Zealand White rabbits were selected and underwent four immunizations. For the first immunization, the positive group received the rEtMIF protein was emulsified with complete Freund’s adjuvant (CFA, Sigma, St Louis MO, USA) at a 1:1 volume ratio using a homogenizer (FLUKO, Shanghai, China) on ice, whereas the negative group was immunized with PBS emulsified with CFA. Subsequent immunizations for both groups employed incomplete Freund’s adjuvant (IFA) instead of CFA. All emulsifications were performed at a 1:1 volume ratio. Subcutaneous immunizations were performed bilaterally along the spine at a dose of 200 µg per rabbit, with a total of four immunizations. Before each immunization, 30 µL of blood was collected from the ear vein, serum was isolated, and antibody titers were determined. The interval between immunizations was 14 days. Seven days after the final immunization, blood was collected via cardiac puncture, incubated at 37°C for 1 h followed by overnight incubation at 4°C, and centrifuged at 3000 rpm for 10 min to collect the serum. The collected serum was heat-inactivated at 56°C for 30 min and stored at −80°C until use. Serum from uninfected rabbits was collected simultaneously as a negative control.

### 2.6 Indirect immunofluorescence assay for localization of EtMIF protein in *E. tenella* merozoites

Indirect immunofluorescence assay was performed according to the method described by Wang et al. (18). The collected *E. tenella* merozoites were smeared onto glass slides and fixed with 4% paraformaldehyde (Sangon Biotech, Shanghai, China) at room temperature for 10 min. After fixation, the samples were permeabilized with 1% Triton X-100 (Beyotime, Shanghai, China) at room temperature for 10 min. Slides were washed three times with PBST (Solarbio, Beijing, China), with 5-min incubations between washes. Samples were blocked with 5% bovine serum albumin (BSA; Beyotime, Shanghai, China) at 37°C for 2 h. The polyclonal antibody against rEtMIF and rabbit negative serum (both diluted 1:800 in PBST) were applied and incubated overnight at 4°C. After washing the slides three times with PBST, the samples were incubated with FITC-labeled goat anti-rabbit IgG secondary antibody (1:5000 dilution in PBST; Abmart, Shanghai, China) at 37°C in the dark for 40 min. Subsequently, the slides were stained with Hoechst 33342 (Beyotime, Shanghai, China) at room temperature for 10 min, washed three times with PBST with 5-min incubations between washes, mounted with an anti-fade mounting medium, and observed under an inverted fluorescence microscope.

### 2.7 Animal experiment

**Figure 1:**
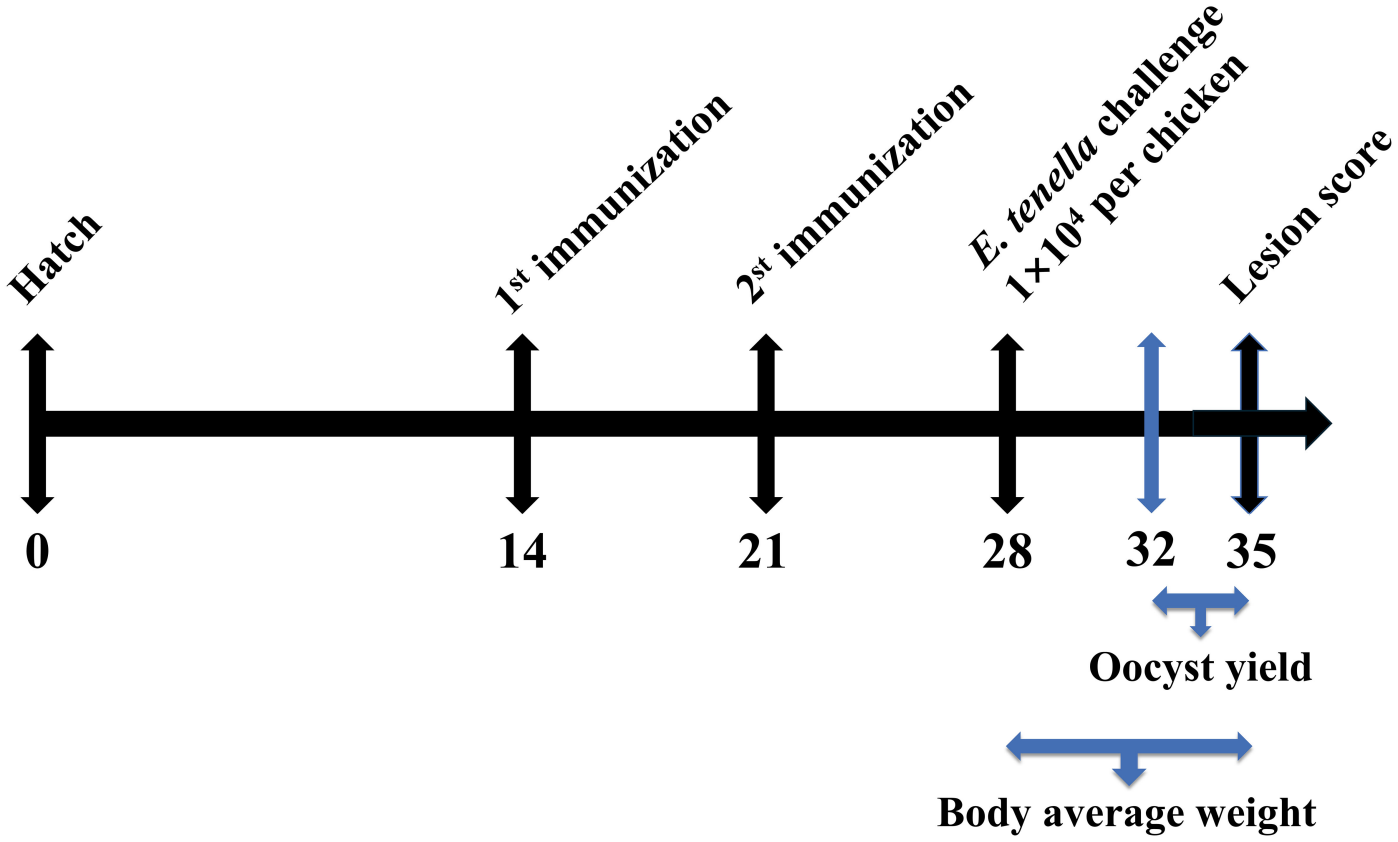
Animal immunization test stage. SPF White Leghorns were subcutaneously immunized with rEtMIF protein on days 14 and 21. On day 28, all chickens (except those in the unchallenged control group) were orally challenged with 1 × 10^4^ sporulated *E. tenella* oocysts per bird. Oocyst shedding was assessed from days 32 to 35, and body weight was recorded on days 28 and 35.

One hundred 1-day-old SPF chickens were selected. Chickens with similar body weights were randomly allocated into five groups (n = 20 per group), with each group further divided into two cages. As shown in **Figure 1**, at 14 and 21 days of age, chickens were subcutaneously immunized in the neck with rEtMIF protein emulsified with Montanide™ ISA 71 VG (ISA 71 VG, Seppic, Paris, France) at doses of 50, 100, or 150 µg. The challenged and unchallenged control groups were simultaneously injected with PBS emulsified with ISA 71 VG. All emulsifications were prepared by mixing the adjuvant and rEtMIF protein (in PBS) at a 70:30 weight ratio, and the resulting emulsion was repeatedly passed through a 0.9 G20 injection needle 20 times to ensure uniformity. Seven days later, chickens in the immunized and challenged control groups were orally inoculated with 1×10^4^ sporulated *E. tenella* oocysts, whereas chickens in the unchallenged control group received PBS orally (**Table 1**). Body weights were recorded on days 14, 21, 28 (before challenge), and 35 (7 days post-challenge). On day 35, fecal samples were collected for oocysts counting, ceca were dissected for lesion scoring, and cardiac blood samples were collected.

**Table 1:** The detail of vaccination regimens.

Groups	Immunization	Dose	Challenge
Unchallenged control	PBS+adjuvant	–	–
Challenged control	PBS+adjuvant	–	*E. tenella* sporulated oocysts(1×10^4^)
rEtMIF (50 µg)	rEtMIF protein+adjuvant	50 µg	*E. tenella* sporulated oocysts(1×10^4^)
rEtMIF (100 µg)	rEtMIF protein+adjuvant	100 µg	*E. tenella* sporulated oocysts(1×10^4^)
rEtMIF (150 µg)	rEtMIF protein+adjuvant	150 µg	*E. tenella* sporulated oocysts(1×10^4^)

### 2.8 *In vivo* immunoprotective parameters

*In vivo* immunoprotective parameters of the rEtMIF protein included clinical, pathological, and parasitological parameters. Clinical parameters included weight gain, survival rate, and mortality rate, which were directly recorded and calculated. Pathological evaluation was based on cecal lesion scores ranging from 0 (none) to 4 (severe), assessed independently by three observers according to the criteria described by Johnso (19). Parasitological parameters included oocyst output and anticoccidial index (ACI) (20). Oocyst output was quantified as the number of oocysts per gram (OPG) of cecal content using the McMaster counting technique (21, 22), and the oocyst index was subsequently calculated. The oocyst index was then calculated based on the following OPG ranges: an OPG ≤ 0.1 was scored as 0; 0.1–1.0 as 1; 2.0–5.0 as 10; 6.0–10.0 as 20; and OPG ≥ 11.0 was scored as 40 (23). The ACI served as a comprehensive parameter for evaluating anticoccidial efficacy and was calculated using the following formula: ACI = (relative weight gain rate + survival rate) − (mean lesion score×10−oocyst index). An ACI ≥ 180 indicated good protection, 160 ≤ ACI < 180 indicated moderate protection, 120 ≤ ACI < 160 indicated limited protection, and ACI < 120 indicated no protection.

### 2.9 Detection of Serum Antibodies and Cytokines

At 14, 21, and 28 days of age, three chickens were randomly selected. Blood was collected via cardiac puncture, and serum samples were obtained by centrifugation and stored at −80°C until use. The levels of antigen-specific IgY were evaluated by enzyme-linked immunosorbent assay (ELISA). Briefly, polystyrene 96-well flat-bottom microtiter plates were coated with 100 µL/well of rEtMIF (1 µg/mL) in coating buffer at 37°C for 1 h, followed by overnight incubation at 4°C. After four washes with PBS containing 0.05% Tween 20 (PBST, pH 7.4), the plates were blocked with 1% bovine serum albumin (BSA; Beyotime, Shanghai, China) at 37°C for 1 h. After four additional washes with PBST, sera diluted in 0.1% BSA-PBST were added and incubated at 37°C for 1.5 h. Plates were then washed again, followed by incubation with 100 µL of HRP-conjugated rabbit anti-chicken IgY (1:10000 dilution in PBST; Bioss, Beijing, China) at 37°C for 1 h. After washing, 50 µL/well of TMB substrate (Bioss, Beijing, China) was added and incubated for 20 min. The enzymatic reaction was terminated by adding 50 µL/well of stop solution, and the optical density (OD450) was measured using a microplate reader. All serum samples were tested in triplicate. Serum levels of IL-8, TNF-α, IL-1, and IFN-γ were measured using chicken cytokine ELISA kits (Shanghai Enzyme-linked Biotechnology Co., Ltd., Shanghai, China) according to the manufacturer’s instructions, and absorbance was read at 450 nm using a microplate reader. Data were obtained from three independent experiments.

### 2.10 Statistical analysis

All data were analyzed using SPSS version 22.0 (IBM Corp., Armonk, NY, USA) and expressed as mean ± standard deviation (SD). Statistical significance was evaluated using one-way ANOVA followed by Tukey’s *post hoc* test to assess group differences. Graphs were generated using GraphPad Prism version 5.0 (GraphPad Software, San Diego, CA, USA). Each experiment was repeated at least three times. Differences were considered statistically significant at *P* < 0.05.

## 3 Results

### 3.1 Gene cloning and plasmid construction

**Figure 2:**
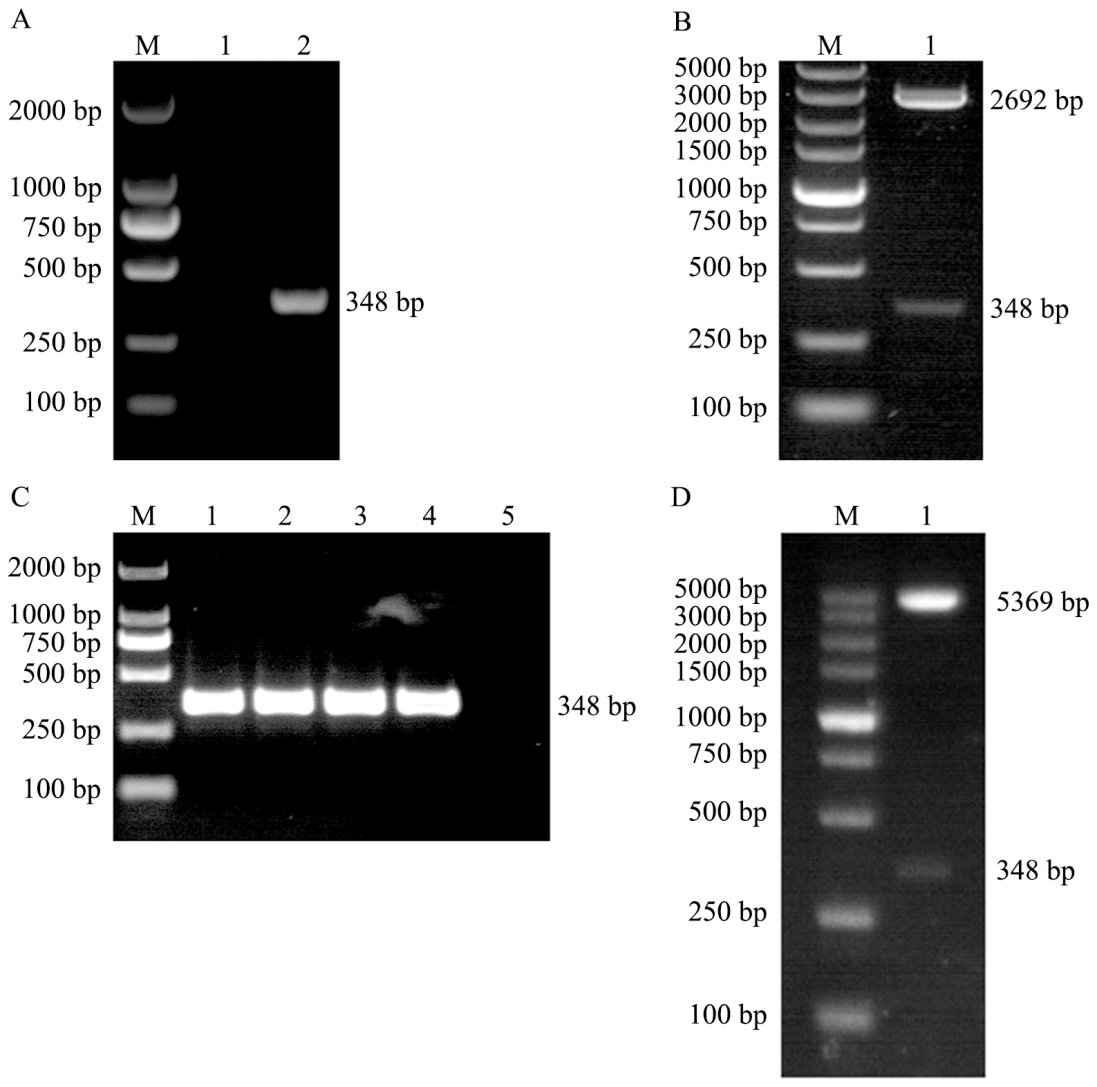
Construction of the pET-28a(+)-EtMIF Expression Vector. **(A)** PCR amplification of the *EtMIF* gene. Lane 1: negative control; lane 2: target band of the *EtMIF* CDS region. **(B)** Double digestion identification of pMD-18T-EtMIF. **(C)** Colony PCR screening results of recombinant pET-28a(+)-EtMIF. Lanes 1–4: target bands of the *EtMIF* CDS region; lane 5: negative control. **(D)** Double digestion identification of recombinant pET-28a-EtMIF. M: DNA marker.

The CDS region of the *EtMIF* gene was amplified by PCR using cDNA as the template, and a specific band of 348 bp was obtained (**Figure 2A**). The PCR product was then cloned into the pMD-18T vector, and positive colonies were selected. Following plasmid extraction and double digestion, bands corresponding to the vector (2692 bp) and the target gene (348 bp) were observed (**Figure 2B**). The target fragment was excised from the pMD-18T vector and subcloned into the pET-28a(+) vector. Positive colonies were screened by colony PCR, followed by double digestion identification and sequencing analysis (**Figures 2C, D**). The prokaryotic expression vector pET-28a(+)-EtMIF was successfully constructed.

### 3.2 Expression and purification of rEtMIF protein

**Figure 3:**
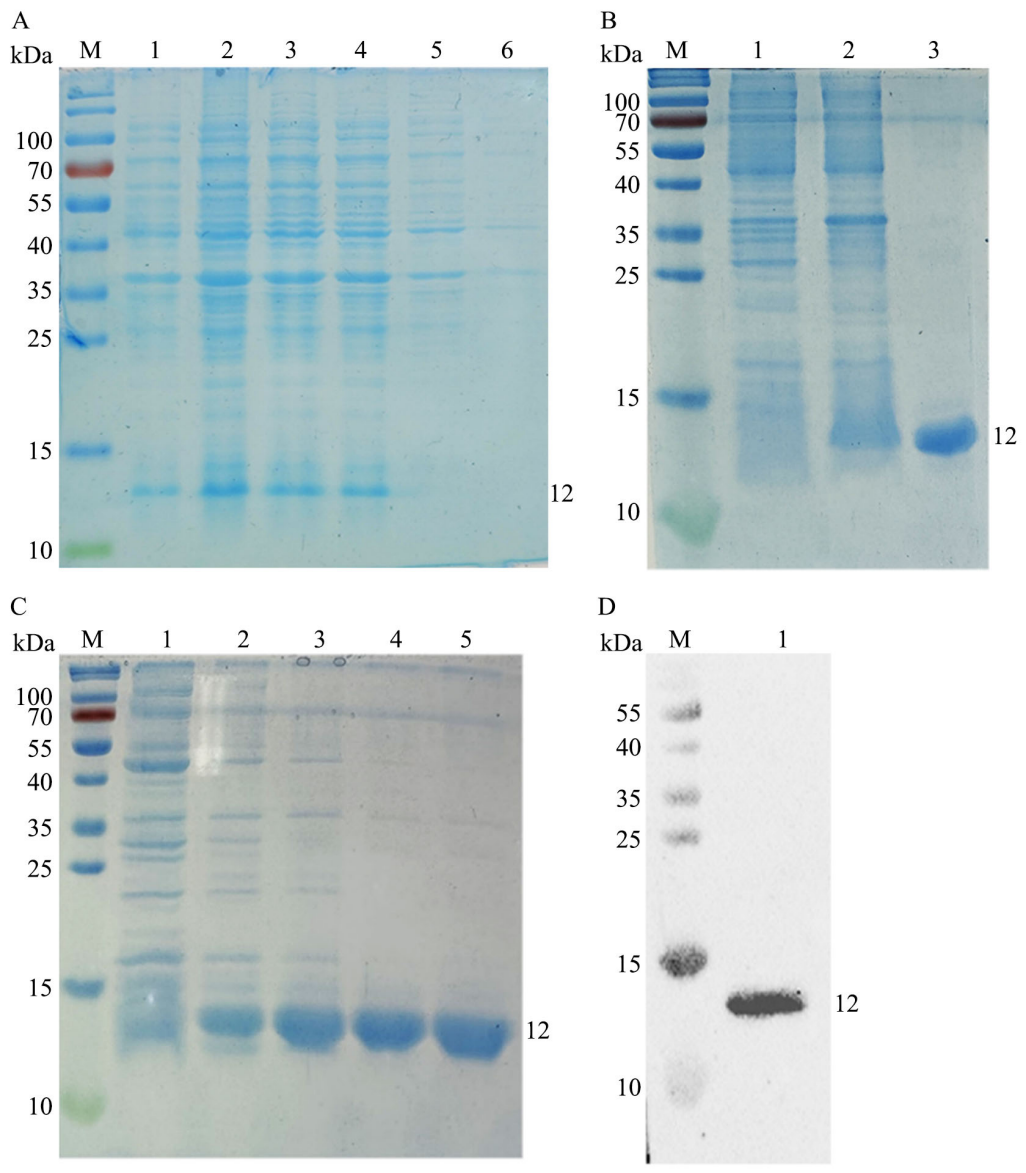
Expression, purification, and identification of rEtMIF. **(A)** SDS-PAGE analysis of rEtMIF expression induced by IPTG at different time points. M: protein marker; lanes 1-4: samples induced with IPTG for 2, 4, 6, and 8 h, respectively; lane 5: uninduced pET-28a(+)-EtMIF control; lane 6: pET-28a(+) empty vector control. **(B)** SDS-PAGE analysis of rEtMIF solubility. Lane 1: pellet of induced pET-28a(+)-EtMIF; lane 2: supernatant of induced pET-28a(+)-EtMIF; lane 3: purified soluble rEtMIF protein. **(C)** SDS-PAGE analysis of rEtMIF eluted with different imidazole concentrations. Lanes 1–5: elution products with imidazole concentrations of 50, 100, 150, 200, and 500 mmol/L. **(D)** Western blot detection of rEtMIF protein using an anti-6×His monoclonal antibody.

After IPTG induction, the expression of rEtMIF protein was analyzed by SDS-PAGE. Coomassie Brilliant Blue staining revealed that rEtMIF was highly expressed after IPTG induction at 37°C for 4 h (**Figure 3A**). After purification, the rEtMIF protein was predominantly present in the soluble fraction, and a target band of approximately 12 kDa was detected (**Figure 3B**). The optimal imidazole elution concentration was determined to be 500 mmol/L (**Figure 3C**). Western blot analysis showed that the rEtMIF protein was specifically recognized by monoclonal antibodies against the 6×His tag, with a target band appearing at approximately 12 kDa, confirming the successful recombinant expression of the rEtMIF protein *in vitro* (**Figure 3D**).

### 3.3 Natural immunogenicity of EtMIF in chickens infected with *E. tenella*

**Figure 4:**
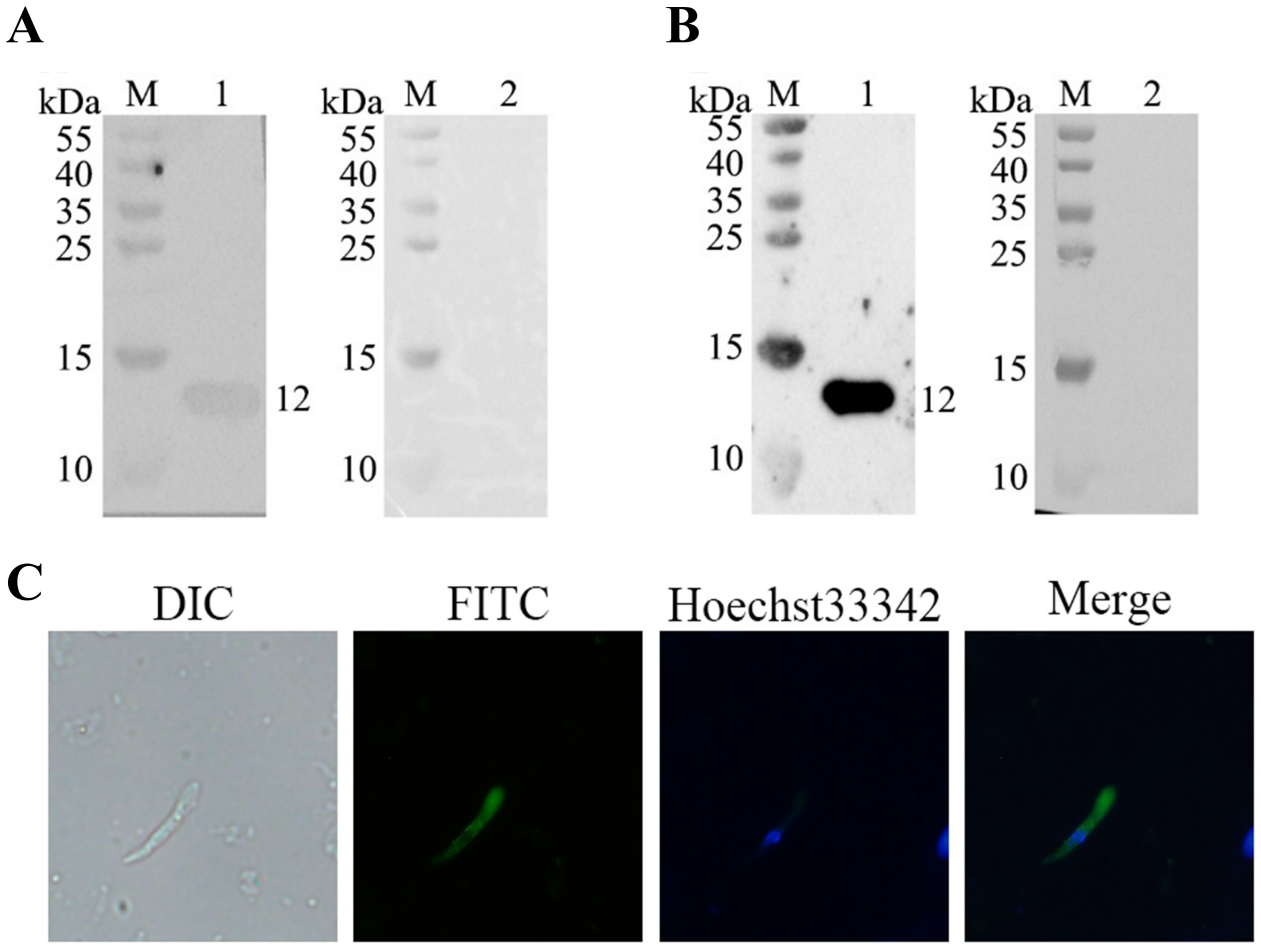
Natural immunogenicity of rEtMIF and localization of EtMIF. **(A)** Detection of rEtMIF protein recognition by *E. tenella*-positive chicken serum. M: protein marker; lane 1: *E. tenella*-positive chicken serum; lane 2: negative chicken serum. **(B)** Detection of rEtMIF protein recognition by rabbit anti-rEtMIF polyclonal antibodies. M: protein marker; lane 1: rabbit anti-rEtMIF polyclonal serum; lane 2: negative rabbit serum. **(C)** Indirect immunofluorescence localization of EtMIF protein in *E. tenella* merozoites (400×).

Western blot analysis showed that the recombinant rEtMIF protein was recognized by *E. tenella*-positive chicken serum (**Figure 4A**) and by rabbit anti-rEtMIF polyclonal antibodies (**Figure 4B**). A specific band of approximately 12 kDa was observed, while no reactivity was detected with negative control sera from chickens and rabbits. These results indicate that EtMIF is a native antigen of *E. tenella* and a promising candidate for vaccine development. Indirect immunofluorescence assay (IFA) revealed that EtMIF was localized both on the surface and within the cytoplasm of *E. tenella* merozoites (**Figure 4C**).

### 3.4 *In vivo* immunoprotective effect of rEtMIF protein

In the challenged control and immunized groups, bloody stools were observed, along with lethargy, reduced appetite, and ruffled feathers. Compared to the challenged control group, the immunized groups exhibited alleviated clinical symptoms. No clinical symptoms were observed in the unchallenged (blank) group. No deaths occurred in any of the experimental groups.

There were no significant differences in average body weight among the groups before the challenge, indicating that immunization with rEtMIF had no adverse effect on chicken growth. On day 7 post-infection, the 50 µg rEtMIF immunized group showed significantly higher weight gain compared to the challenged control group (*P* < 0.01), while the 100 µg group also showed significantly higher weight gain (*P* < 0.05). No significant difference was observed between the 150 µg rEtMIF group and the challenged control group (**Table 2**).

**Figure 5:**
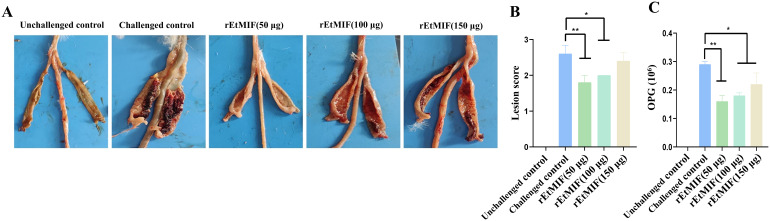
Effects of different doses of rEtMIF on cecal lesion severity in chickens. **(A)** Macroscopic anatomy of the cecum. **(B)** Cecal lesion scores. **(C)** OPG. Note: “*” denotes significant difference (*P* < 0.05), “**” denotes highly significant difference (*P* < 0.01).

Similarly, the cecal lesion score of the 50 µg rEtMIF group was significantly lower than that of the challenged control group (*P* < 0.01), and the 100 µg rEtMIF group also showed a significant reduction in lesion scores (*P* < 0.05). No significant difference in lesion scores was observed in the 150 µg rEtMIF group (**Table 2**, **Figures 5A, B**).

**Table 2:** Summary of immunization outcomes.

Groups	Relative body weight gain rate(%)	Oocyst index	Cecum Mean Lesion value	Anticoccidial Index(ACI)
Unchallenged control	100.00**	0	0.00	200.00
Challenged control	45.44	1	26.00**	118.44
rEtMIF 50 μg	80.48**	1	18.00	161.48
rEtMIF 100 μg	66.31*	1	20.00*	145.31
rEtMIF 150 μg	56.41	1	24.00*	131.41

“*” denotes significant difference (*P* < 0.05), “**” denotes highly significant difference (*P* < 0.01).

Compared to the challenged control group, chickens immunized with rEtMIF protein exhibited a significant reduction in oocyst output (*P* < 0.05) (**Figure 5C**). Notably, the anticoccidial index (ACI) of the 50 µg rEtMIF immunized group exceeded 160, whereas the ACI values of the 100 µg and 150 µg groups remained below 160. These findings indicate that the 50 µg dose provided moderate protective efficacy and represented the optimal immunization dose among the three tested doses against *E. tenella* infection.

### 3.5 Levels of serum antibodies

**Figure 6:**
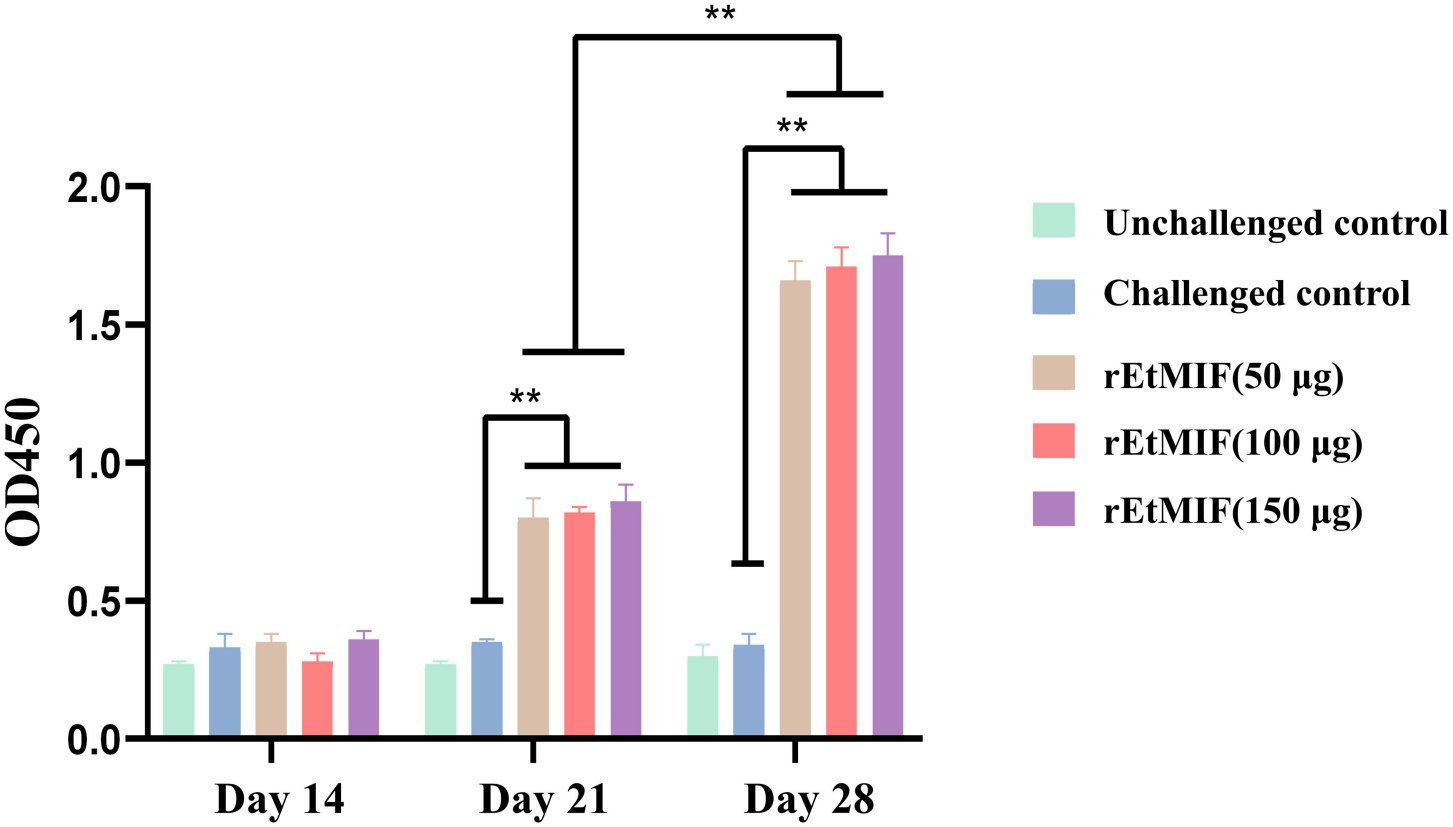
Serum antibody IgY levels in chickens at different time points (days 14, 21, and 28). Chickens were divided into five groups: unchallenged control, challenged control, and three rEtMIF-immunized groups (50, 100, and 150 μg) (n = 3 per group) Note: “*” denotes significant difference (*P* < 0.05), “**” denotes highly significant difference (*P* < 0.01).

Seven days after the first immunization, the levels of IgY antibodies in the serum of the rEtMIF immunized groups at different doses were significantly higher than those in the challenged control group (*P* < 0.05), with the 150 µg rEtMIF group exhibiting the highest antibody levels. Seven days after the booster immunization, the IgY antibody levels in the rEtMIF immunized groups remained significantly higher than those of the challenged control group, and antibody levels after the booster immunization were significantly higher than after the primary immunization (*P* < 0.05, **Figure 6**). These results demonstrate that rEtMIF is a native antigen of *E. tenella* capable of eliciting a strong humoral immune response to combat *E. tenella* infection.

### 3.6 Expression of cytokines

**Figure 7:**
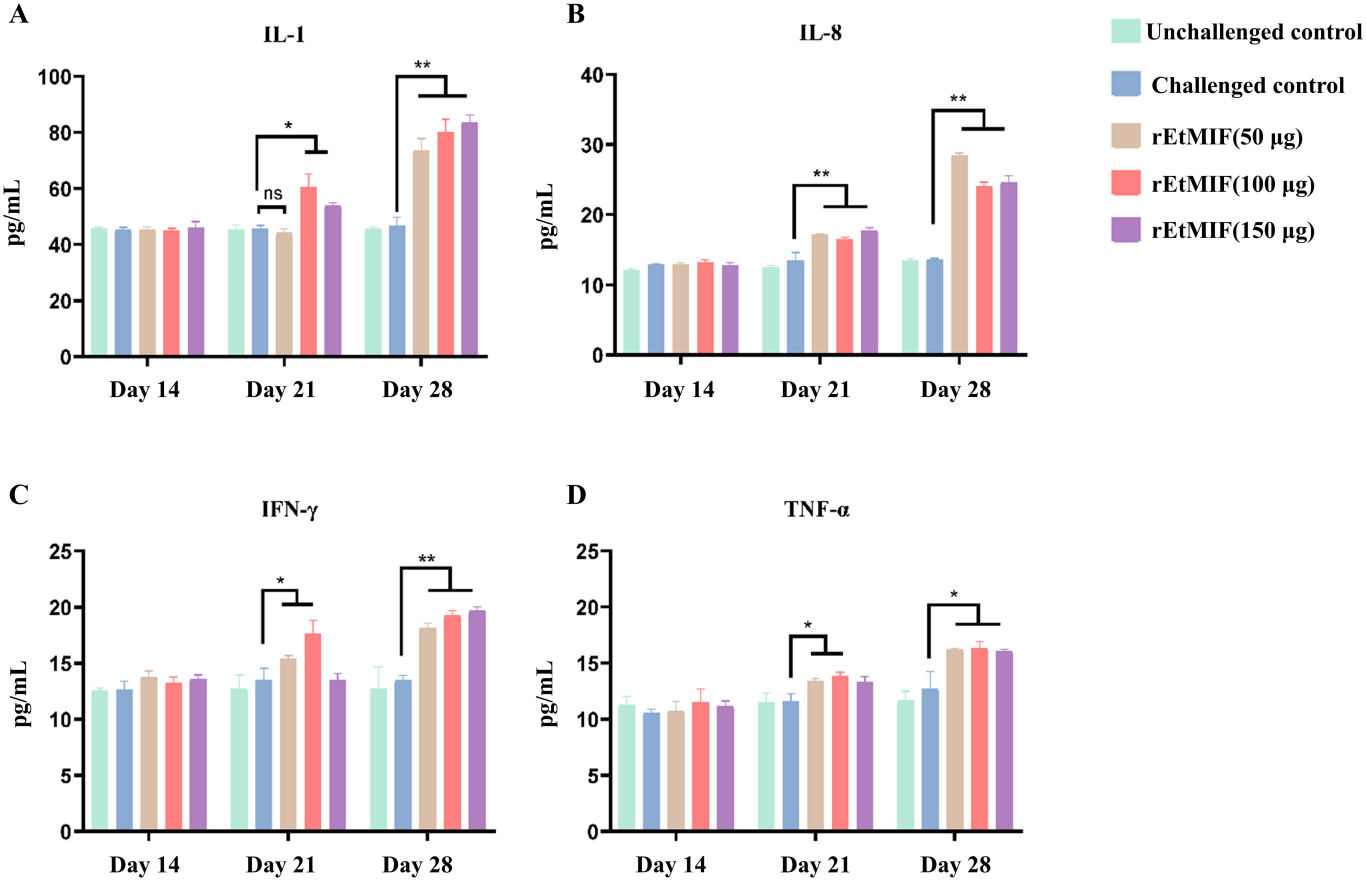
Serum cytokine levels in chickens at different time points (days 14, 21, and 28). **(A)** Expression of IL-1; **(B)** IL-8; **(C)** IFN-γ; **(D)** TNF-α. Chickens were divided into five groups: unchallenged control, challenged control, and three rEtMIF-immunized groups (50, 100, and 150 μg) (n = 3 per group). “*” denotes significant difference (*P* < 0.05), “**” denotes highly significant difference (*P* < 0.01), “ns” denotes highly significant difference (*P* > 0.05).

Serum levels of IL-1, IL-8, TNF-α, and IFN-γ were measured using ELISA kits. No significant differences were observed among groups before immunization. Prior to the booster immunization, IL-1 levels were significantly higher in the 100 µg and 150 µg rEtMIF immunized groups compared to the challenged control group (*P* < 0.05), while IFN-γ and TNF-α levels were significantly higher in the 50 µg and 100 µg rEtMIF groups (*P* < 0.05). Additionally, IL-8 levels were significantly higher in all rEtMIF immunized groups compared to the challenged control group (*P* < 0.01). Before challenge, the levels of IL-1, IL-8, and IFN-γ in each rEtMIF immunized group were significantly higher than those in the challenged control group (*P* < 0.01), and TNF-α levels were also significantly higher (*P* < 0.05) (**Figure 7**).

## 4 Discussion

*E. tenella* is the most prevalent and pathogenic intracellular parasite causing chicken coccidiosis, a disease that severely impacts poultry health and production. Recent reports indicate incidence rates of 50%-70%, with mortality rates exceeding 80% in severe outbreaks (24, 25). Although anticoccidial drugs and commercial vaccines, including live attenuated and inactivated formulations, are widely used, issues such as drug resistance, drug residues in poultry products, and the risk of pathogenic reversion limit their long-term effectiveness (26). Consequently, there is an urgent need to develop safe, effective, and sustainable vaccines for the prevention and control of chicken coccidiosis. Recombinant protein vaccines are increasingly recognized for their ability to elicit antigen-specific antibody responses and robust humoral immunity. In this context, we successfully expressed, purified, and evaluated the recombinant rEtMIF protein as a potential vaccine candidate against *E. tenella* infection.

MIF is a highly conserved cytokine originally identified in delayed-type hypersensitivity studies (27). It regulates immune responses by inhibiting macrophage migration, promoting inflammatory cell aggregation, enhancing phagocytic activity, and stimulating the secretion of pro-inflammatory cytokines such as TNF-α and IL-1 (28). Given these biological functions, MIF homologs from parasitic organisms have attracted attention as promising vaccine targets. In this study, high-titer polyclonal antibodies against rEtMIF were successfully generated in rabbits, and specific reactivity was observed with sera from both rEtMIF-immunized rabbits and chickens naturally infected with *E. tenella*. Immunofluorescence localization revealed that rEtMIF was distributed both on the surface and within the cytoplasm of merozoites, consistent with previous reports showing upregulated MIF expression during the schizogony and gametocyte stages (29). These findings indicate that EtMIF is an endogenous antigen expressed at critical developmental stages, accessible to host immune recognition.

The immunization-challenge trials further demonstrated the protective efficacy of rEtMIF. Chickens immunized with 50 µg of rEtMIF exhibited significantly greater weight gain, lower cecal lesion scores, and reduced oocyst shedding compared to challenged controls (*P* < 0.05), suggesting moderate anticoccidial protection. However, this level of protection is not considered good protection, indicating that using the current rEtMIF alone may not be optimal or may have inherent limitations. Therefore, future studies should explore combining this immunization with other vaccines or treatment strategies to achieve improved protective efficacy. Although chickens immunized with higher doses (100 µg and 150 µg) also showed partial protection, the 50 µg dose provided the best balance between immune response and cost-effectiveness. These observations are consistent with previous studies on MIF homologs in other parasitic models. For instance, recombinant TgMIF immunization significantly prolonged survival and reduced brain cyst burdens in *Toxoplasma gondii* infection models (30). Overexpression of MIF in *Plasmodium yoelii* attenuated parasite virulence and modulated host immunity (31), while EaMIF DNA vaccines demonstrated significant protection against *Eimeria* acervuline (32). Collectively, these findings reinforce the immunoprotective potential of parasite-derived MIFs and support the feasibility of developing rEtMIF as a subunit vaccine for coccidiosis control.

The immune protection conferred by rEtMIF immunization appears to involve both cellular and humoral immune responses. Notably, significant increases in serum IFN-γ, IL-8, and TNF-α levels were observed in the rEtMIF-immunized groups prior to challenge (*P* < 0.01). IFN-γ plays a key role in macrophage activation and Th1-type immune responses (33), IL-8 recruits neutrophils and promotes tissue repair (34), while TNF-α enhances phagocytic activity (35), collectively contributing to early parasite elimination. IgY plays a key role in humoral immunity against coccidial infection, providing long-term protection and supporting the formation of immune memory (36). ELISA analysis revealed that rEtMIF vaccination significantly elevated antigen-specific IgY antibody levels both after the primary and booster immunizations (*P* < 0.01), highlighting the importance of humoral immunity in protection against *E. tenella*.

Interestingly, a dose-response paradox was observed in our study: higher antigen doses of rEtMIF (100 µg and 150 µg) failed to confer superior protection compared to the 50 µg dose and even resulted in reduced efficacy. This nonlinear pattern suggests that the immune protection induced by rEtMIF does not follow a simple dose-dependent escalation, but is instead governed by immune regulatory thresholds. Effective vaccine responses rely on a delicate balance between immune activation and regulation. While robust pro-inflammatory responses are essential for resolving *E. tenella* infection, excessive immune stimulation can trigger regulatory mechanisms that limit effector function or induce immune tolerance. For instance, in malaria infection, immune checkpoints such as PD-1 and CTLA-4 suppress T cell proliferation and inhibit IFN-γ production via distinct signaling pathways, thereby preventing immunopathology such as cerebral malaria (37). Similarly, Elliott et al. demonstrated that malaria-derived components suppress dendritic cell (DC) maturation in a strictly dose-dependent manner, independent of live parasites (38), suggesting a direct role for parasite-derived molecules. Drawing a parallel, we speculate that high-dose rEtMIF may similarly suppress immune responses by overwhelming the functional capacity of DCs or other antigen-presenting cells, thereby contributing to the reduced protective efficacy observed at higher immunization doses. Additionally, a biphasic pattern has been observed in DC-to-parasite ratios: low ratios promote activation, while high ratios suppress DC function (39).

MIF, a non-classically secreted pro-inflammatory cytokine, plays a multifaceted role in immune regulation. It enhances TLR4 expression, activates the ERK1/2 signaling pathway, inhibits p53-mediated apoptosis, and counteracts glucocorticoid-induced immunosuppression. During the early phase of infection, MIF contributes to pathogen clearance; however, excessive MIF expression may lead to uncontrolled inflammation, exacerbating tissue damage and promoting the development of sepsis and ARDS (40). This hypothesis is further supported by evidence from other disease models. Aharon et al. reported that high-expression MIF genotypes (e.g., CATT7 and -173C) are associated with exacerbated acute graft-versus-host disease (GVHD) and glucocorticoid resistance in HSCT patients, likely due to sustained pro-inflammatory signaling and impaired immune regulation (41). Zhou et al. (42) showed that *Toxoplasma gondii*-derived TgMIF aggravates liver inflammation by inducing hepatocyte pyroptosis via NLRP1 inflammasome activation and Gasdermin D upregulation. Based on these findings, we hypothesize that excessive rEtMIF may similarly amplify inflammatory responses, impair protective immunity, and contribute to the reduced efficacy observed at higher antigen doses. Zhu et al. showed that MIF enhances the release of pro-inflammatory extracellular vesicles (EVs) through the CD74-ERK1/2-Myc signaling axis in colorectal cancer cells, thereby remodeling the immune microenvironment and sustaining inflammation-driven progression (39). The 50 µg dose may represent a functional threshold at which immunogenicity is maximized without triggering negative feedback regulation. Further mechanistic studies are warranted to validate this hypothesis, particularly focusing on dendritic cell signaling pathways, regulatory T cell activation, cytokine receptor desensitization, and antigen tolerance mechanisms.

Our findings demonstrate that recombinant rEtMIF protein induces robust cellular and humoral immune responses, conferring partial protection against *E. tenella* infection. Nevertheless, the level of protection achieved with rEtMIF alone remains moderate, indicating that combining rEtMIF with other key antigens may be necessary to achieve broader and more durable immunity. Accumulating evidence from other *Eimeria* antigen studies, such as rEtSAG4, rEtAN1-ZnFP, and rEmARM-β (43–45), further supports this strategy. In conclusion, we successfully cloned and expressed the *EtMIF* gene, validated its immunogenicity and partial protective efficacy, and proposed it as a promising subunit vaccine candidate for the prevention and control of chicken coccidiosis. Future work will focus on elucidating the detailed immune mechanisms mediated by rEtMIF and exploring its synergistic potential in multi-antigen vaccine formulations.

## 5 Conclusions

In this study, we successfully constructed the prokaryotic expression vector of *E. tenella* MIF, purified the recombinant protein, and localized it via immunofluorescence in *E. tenella* merozoites. Our research demonstrated that low doses of rEtMIF effectively triggered specific immune responses, and vaccinated chickens exhibited partial protection against *E. tenella* infection. Therefore, rEtMIF should be considered a promising vaccine candidate against *E. tenella*. Complete protection may be achieved by combining EtMIF with other immunogenic antigens to enhance immunity against *E. tenella* challenge.

## Data Availability

The original contributions presented in the study are included in the article/supplementary material. Further inquiries can be directed to the corresponding author.
